# Polarization‐Multiplexed Metalens Enables Switchable and Compact Chromatic Confocal Sensing with Dual‐Mode Precision Control

**DOI:** 10.1002/advs.202517093

**Published:** 2025-11-12

**Authors:** Zhicheng Zhao, Yuting Jiang, Tao Lai, Wenxiang Peng, Yueqiang Hu, Shanyong Chen

**Affiliations:** ^1^ College of Intelligence Science and Technology National University of Defense Technology Changsha 410073 China; ^2^ College of Mechanical and Vehicle Engineering Hunan University Changsha 410082 China

**Keywords:** 3D measurement, chromatic confocal measurement, metasurface

## Abstract

Chromatic confocal measurement, as a non‐contact, rapid, and high‐precision optical technique, holds significant applications in industrial manufacturing, biomedical imaging, and ultraprecision metrology. However, conventional implementations relying on refractive lens assemblies suffer from inherent limitations in size, weight, and functional flexibility, limiting compatibility across diverse scenarios. Metalens, as a planar, ultrathin, and multifunctional optical element, exhibits inherent dispersion advantages. Here, conventional design paradigms are broken by leveraging a polarization‐multiplexed metasurface to create the first chromatic confocal sensor with dynamically switchable measurement modes, achieving 10‐fold miniaturization (Ø1 mm). The sensor not only offers significant advantages such as lightweight and integration, but also provides additional measurement modes, including high‐accuracy and extended‐range modes. Following the tests of system, measurement range of 400 µm and 1.57 mm, axial accuracy of ± 0.25 and ± 1.45 µm are achieved, under orthogonal polarized illumination within a 500–700 nm operating wavelength range, respectively. Further, to verify the performance of measurement system facing different measurement requirements, the three‐dimensional (3D) topography of a concave spherical mirror and the thickness of transparent glass sheet are measured successfully. The novel chromatic confocal sensor demonstrates great potential for practical optical metrology and establishes a new design framework for adaptive multi‐functional metrology systems.

## Graphical abstract

This work breaks conventional design paradigms by leveraging a polarization‐multiplexed metasurface to create the first chromatic confocal sensor with dynamically switchable measurement performance, achieving 10‐fold miniaturization (Ø1 mm vs cm‐scale conventional probes). The sensor not only offers significant advantages such as lightweight and integration, but also provides additional measurement modes, including high‐accuracy and extended‐range modes. It can be widely applied in ultraprecision measurement fields.

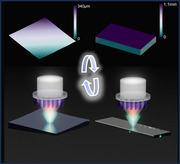

## 1 Introduction

Optical 3D metrology, leveraging the amplitude, polarization, and phase characteristics of light fields, has emerged as a critical non‐contact measurement technique with broad applications spanning industrial quality control,^[^ ^1^ ^]^ biomedical imaging^[^ ^2^, ^3^ ^]^ and ultraprecision measurement engineering.^[^ ^4^, ^5^ ^]^ The predominant optical 3D metrology approaches include interferometry techniques (e.g., white‐light interferometry,^[^ ^6^ ^]^ differential interference contrast^[^ ^7^ ^]^), structured light projection (e.g., laser triangulation,^[^ ^8^ ^]^ Moiré topography^[^ ^9^ ^]^), and confocal microscopy (e.g., laser confocal,^[^ ^10^ ^]^ chromatic confocal^[^ ^11^ ^]^). Interferometric techniques achieve nanometer‐level resolution by extracting phase information from interference patterns. However, these systems typically require complex optical configurations with stringent environmental stability. Furthermore, their limited field of view necessitates image stitching for large‐area measurements, introducing computational complexity in data processing. Structured light projection techniques enable rapid large‐area measurements, but its accuracy is limited to the sub‐millimeter range due to factors such as the spatial density of projected patterns and baseline distance. Confocal techniques rely on intensity‐based depth discrimination at the focal plane, offering sub‐micrometer accuracy. By integrating high‐precision multi‐axis scanning stages, confocal systems enable rapid, robust and high‐ precision 3D profiling, making them indispensable for real‐time inspection in dynamic environments. In terms of the trade‐off between high precision and measurement efficiency, chromatic confocal approach provides distinct advantages over laser confocal method by eliminating mechanical z‐axis scanning.

In contrast to achromatic engineering,^[^ ^12^ ^]^ chromatic confocal measurement (CCM) intentionally utilizes longitudinal chromatic aberration as a functional mechanism. In this technique, broadband illumination generates wavelength‐dependent focal planes distributed axially, establishing a precise wavelength‐to‐depth correlation that effectively creates a chromatic “ruler.” The performance of CCM systems is primarily characterized by two key parameters: the chromatic range and axial resolution. Traditional refractive implementations face fundamental limitations – the high Abbe number of optical glasses. A chromatic confocal probe necessitates complex multi‐lens assemblies to achieve sufficient chromatic dispersion, resulting in bulky configurations with stringent alignment requirements. Commercial chromatic confocal sensors typically feature outer diameters in the range of several centimeters. Diffractive optical elements (DOEs) inherently exhibit strong dispersion. A chromatic confocal sensor (CCS) based on a single‐layer phase‐type Fresnel zone plate with a diameter of 11.27 mm has been demonstrated, achieving a 16 mm dispersion range and 0.8 µm axial resolution.^[^ ^13^ ^]^ However, its low numerical aperture (NA) of 0.11 restricts the maximum measurable tilt angle. Additionally, Fresnel zone plates suffer from higher‐order diffraction effects, and their small feature sizes pose fabrication challenges, hindering miniaturization and system integration.

Recent advances in optical metasurfaces – a novel diffractive element – have opened new possibilities for 3D optical metrology. These subwavelength‐structured planar devices enable unprecedented control over the phase,^[^ ^14^ ^]^ polarization^[^ ^15^ ^]^ and frequency^[^ ^16^ ^]^ of light, offering significant advantages over conventional diffractive optics: higher diffraction efficiency, reduced optical noise,^[^ ^17^ ^]^ and ultrathin form factor that facilitates system integration with multifunctions.^[^ ^18^ ^]^ Currently, several applications have been explored for integrated metasurface‐based 3D optical measurement systems, including holography,^[^ ^19^, ^20^ ^]^ shear interferometry,^[^ ^21^, ^22^, ^23^, ^24^, ^25^, ^26^, ^27^ ^]^ intensity transfer equations,^[^ ^28^, ^29^, ^30^ ^]^ and wavefront sensing.^[^ ^31^, ^32^ ^]^ Regarding dispersion confocal measurement, a compact confocal sensor based on metasurfaces has been proposed. This system employs a coaxial structure and achieves a dispersion range of 300 µm with an axial resolution better than 1 µm over a working wavelength range of 540–720 nm.^[^ ^33^ ^]^ To enhance scanning speed, a line‐dispersion confocal measurement system based on metasurfaces has also been demonstrated. This system utilizes an off‐axis configuration, achieving a dispersion range of 1.56 mm within a working wavelength range of 800–1000 nm, with a measurement accuracy of 20 µm.^[^ ^34^ ^]^ However, a limitation of the aforementioned CCM systems is that their performance remains fixed and unchangeable. For different measurement scenarios, it necessitates redesigning and customizing the metasurface, which subsequently increases costs. Future progress in this field will require developing switchable or reconfigurable metasurface platforms to overcome this critical challenge.

In this study, we propose for the first time a compact chromatic confocal sensor based on polarization‐multiplexed metalens (CCS‐ML) enabling switchable measurement performance, which features high‐accuracy and extended‐range modes, namely x‐polarized (x‐pol) and y‐polarized (y‐pol) modes. The sensor probe employs a coaxial structure that includes a parabolic reflective mirror and a single‐layer metasurface consisting of silicon nitride (SiN) nanofins with a diameter of 1 mm, replacing the complex lens assemblies traditionally found in sensor probes. SiN exhibits weaker structural dispersion and a lower extinction coefficient, which contributed to better lens focusing capability. Under x polarization light incidence, the sensor with high‐NA (0.45) achieves a dispersion range of 400 µm, high accuracy of ± 0.25 µm, and the optimal axial resolutions of 70 nm. Under y polarization light incidence, the sensor with low‐NA (0.125) extends the dispersion range to 1.57 mm by leveraging non‐uniform intensity modulation of the light source, while maintaining an axial accuracy of ± 1.45 µm, and an optimal axial resolution of 0.3 µm. Both modes operate within the same wavelength range of 500–700 nm. Additionally, we experimentally measured the 3D topography of a standard step block, a concave spherical reflective mirror, and the thickness of a glass slide, validating the high performance of our sensor. Balancing measurement range, maximum measurable tilt angle, and resolution, we can switch between two different performance modes sensors simply by changing the polarization state of the incident light for different measurement requirements. This sensor is closely aligned with the demands of real‐world measurement engineering. It not only reduces operational costs and complexity for users but also embodies a paradigm shift from conventional “one‐device‐one‐function” systems toward adaptive, multi‐functional optoelectronics. Our research paves the way for utilizing metasurfaces to develop compact, lightweight, and multifunctional chromatic confocal sensors, which can be widely applied in ultraprecision measurement fields.

## 2 Results and Discussion

### 2.1 Chromatic Confocal Sensor Probe Framework

**Figure 1:**
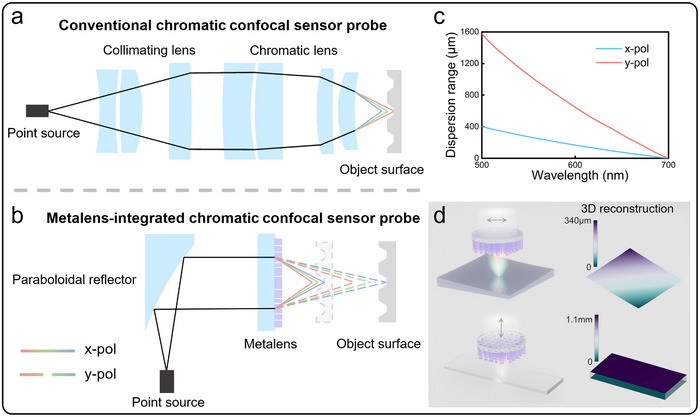
a) Schematic diagram of the conventional chromatic confocal probe. b) Schematic diagram of Metalens‐integrated chromatic confocal probe. c) Displacement range of chromatic confocal sensor based on polarization multiplexing metalens (CCS‐ML) in x‐pol and y‐pol modes with working wavelength range from 500–700 m. d) Schematic diagram of 3D topography measurement application by CCS‐ML x‐pol and y‐pol modes.

The conventional chromatic confocal probe configuration is illustrated in **Figure** **1**a, comprising two essential optical components: an achromatic collimating lens assembly that transforms a white‐light point source into a collimated beam, and a chromatic dispersion lens assembly that focuses parallel beam while introducing controlled axial chromatic dispersion. For a single refractive lens under collimated illumination, the axial dispersion length *L_ch_* =  *f*/ν, where *f* is the focal length at design wavelength and ν is the Abbe number of the lens material. Typical crown or flint glasses (ν > 20) require to combine multiple refractive lenses to optimize for large axial dispersion length, resulting in bulky, alignment‐sensitive systems. In contrast, diffractive lenses exhibit fundamentally different dispersion characteristics. Their effective Abbe number can be approximated as ν  = λ_*d*_ /(λ_*F*_ −  λ_*C*)_ =   − 3.45, where the subscripts *d*, *F*, and *C* represent three pre‐determined wavelengths. For the visible wavelength range, they are 587, 486, and 656 nm. It is significantly lower than the Abbe number of the refractive lens, enabling significant axial dispersion with just a single element. Capitalizing on this property, our design replaces both conventional lens groups with a parabolic mirror and a ultrathin single‐layer 1‐mm‐diameter metalens, achieving more compact and lightweight, as shown in Figure 1b. Notably, our sensor incorporates a polarization dimension of light, enabling switchable performance. Figure 1c shows two distinct dispersion ranges can be independently generated separately, through utilizing orthogonal linearly polarized light. Figure 1d illustrates typical measurement applications, x‐polarized (x‐pol) mode is ideal for high‐precision measurement of high‐slope surfaces like spherical reflective mirrors, while y‐polarized (y‐pol) mode expands the dispersion range to 1.57 mm for characterizing larger height differences structures such as glass thickness measurements. The two dispersion ranges are deliberately non‐overlapping, which offers the additional convenience of mode selection through simple axial positioning when using random polarization.

### 2.2 Metalens Design

**Figure 2:**
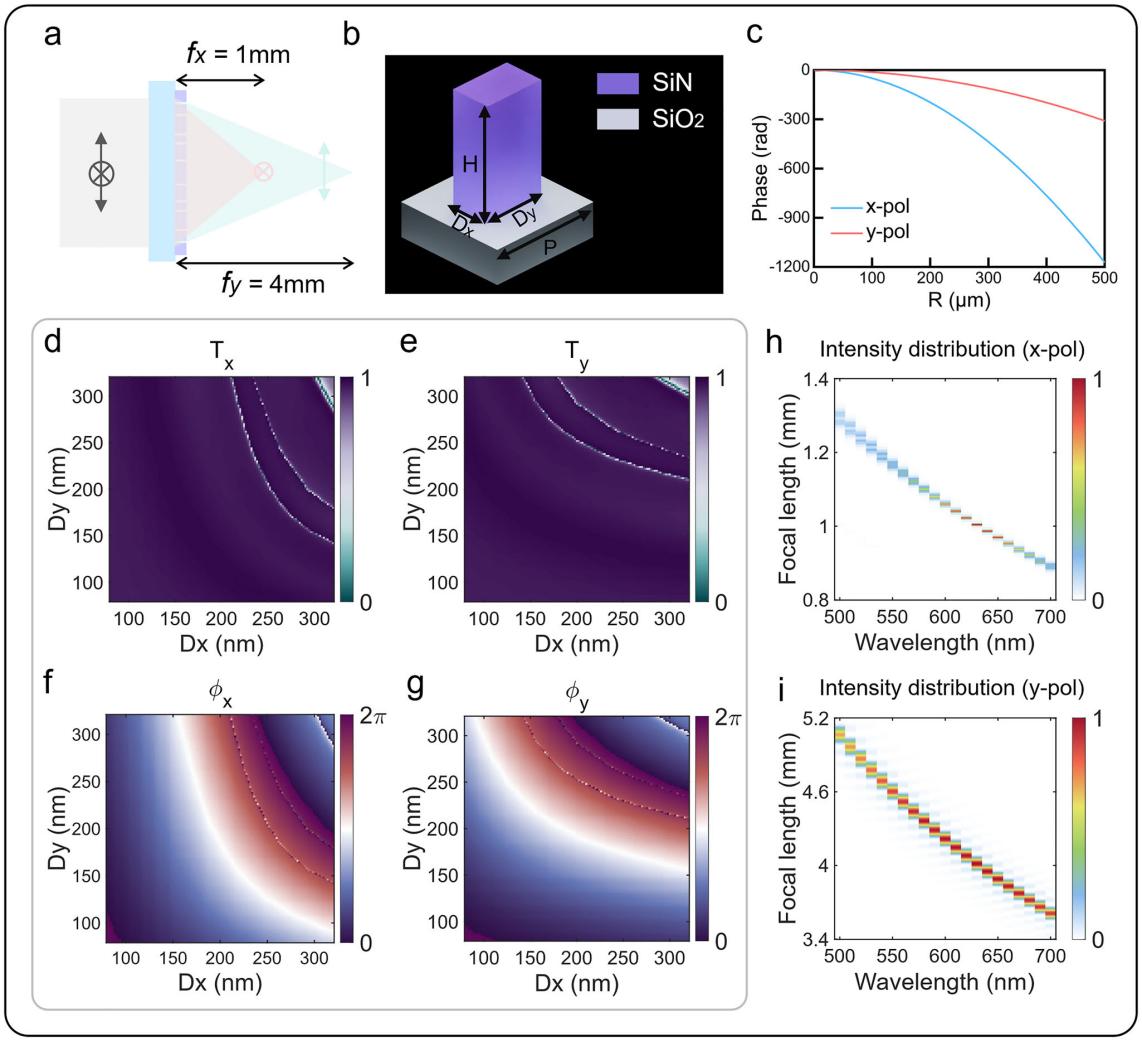
a) Schematic diagram of the polarization multiplexing metalens with diameter of 1 mm, focal length of 1 mm and 4 mm for x‐polarized (x‐pol) and y‐polarized (y‐pol) light at designed wavelength of 632.8 nm. b) Schematic diagram of the meta‐atom composed of silicon nitride (SiN) nanofin arranged on a silicon dioxide (SiO_2_) substrate with 1200 nm height and 400 nm period. c) The phase profile of designed metalens along *x*‐axis for x‐pol and y‐pol. d,e) Simulated transmittance color map of the meta‐atom geometric parameters (Dx and Dy) for x‐pol and y‐pol. f,g) Simulated phase color map of the meta‐atom geometric parameters (Dx and Dy) for x‐pol and y‐pol. h,i) Simulated focal length of metalens for wavelengths ranging from 500 to 700 nm for x‐pol and y‐pol.

The schematic of the designed metalens is shown in **Figure** **2**a. The metalens has a diameter of 1 mm and is designed for a wavelength of 632.8 nm. When x‐polarized light is incident, it exhibits a focal length of 1 mm and a NA of 0.45. In contrast, when y‐polarized light is incident, the focal length increases to 4 mm with a NA of 0.125. The phase distribution of the metalens can be calculated using the following formula:

(1) $$\varphi \left(x,y\right)=-\frac{2\pi }{\lambda }\left(\sqrt{x^{2}+y^{2}+f^{2}}-f\right)$$

where (*x*, *y*) are the spatial coordinates of the metalens, *λ* is the design wavelength, and *f* represents the focal length. Figure 2c illustrates the distinct phase profiles generated under orthogonal linear polarization states. The polarization‐multiplexing functionality is achieved through carefully engineered anisotropic meta‐atoms, whose design is detailed in Figure 2b. Each meta‐atom consists of a silicon nitride (SiN) nanofin with 1200 nm height and 400 nm period, featuring a rectangular cross‐section and arranged on a silicon dioxide (SiO_2_) substrate. The nanofin length (Dx) and width (Dy) can be independently adjusted to control the transmission phase for x‐ and y‐polarized incident light, respectively. The Optical constant of SiN is shown in Figure S1 (Supporting Information). Through finite difference time domain (FDTD) simulations, we obtained the performance of the meta‐atoms under orthogonal linear polarization light, mapping the transmittance and phase responses versus geometric parameters, as shown in Figure 2d–g. Structures with transmittance exceeding 0.5 were selected to match the target phase profiles in Figure 2c through optimization algorithms. To account for the inherent structural dispersion of meta‐atom, we also obtained the transmittance and phase responses of meta‐atom at 10 nm intervals across the 500–700 nm wavelength range. The axial focal distributions of the metalens were calculated using angular spectrum propagation algorithms, as demonstrated in Figure 2h,i. The results successfully met our design objectives. The meta‐atom itself exhibits weak structural dispersion from 500 to 700 nm, as detailed in Section S2 (Supporting Information). The focal point is defined as the position of the maximum intensity, as shown in the 1D plot of Figure S9 (Supporting Information). The chromatic confocal sensor establishes a one‐to‐one correspondence between the focal position and the wavelength. By using a spectrometer to detect the maximum light intensity of the returned signal at a specific position, the optimal focusing wavelength at that location can be determined. This allows the height of the measured object to be derived based on the relationship between wavelength and focal distance.

### 2.3 Metalens Fabrication and Characterization

**Figure 3:**
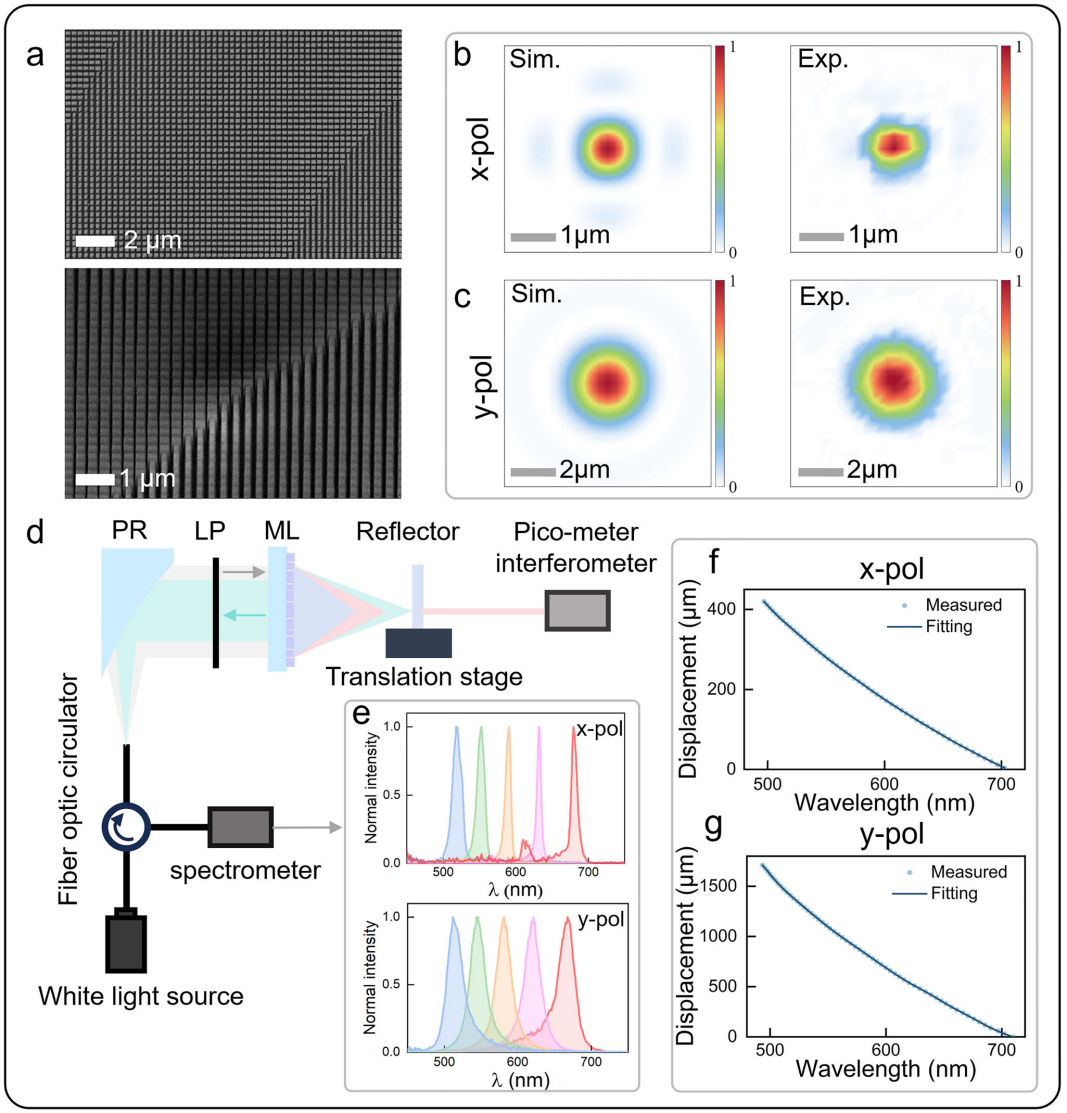
a) Scanning electron microscopy top‐view (top) and side‐view (bottom) image of the fabricated metalens. b,c) Simulated (left) and measured (right) intensity distributions at the focal planes for x‐pol and y‐pol. d) Optical calibration setup for CCS‐ML. PR: parabolic reflective mirror. LP: linear polarizer. ML: metalens. e) The measured spectral signals at five locations for x‐pol (top) and y‐pol (bottom). f) Calibration curve fitting for x‐pol (top) and y‐pol (bottom). The displacement of reflector as a function of peak wavelength.

The metalens was fabricated using standard electron‐beam lithography followed by reactive ion etching processes. **Figure** **3**a presents the scanning electron microscopy top‐view and side‐view images of the fabricated metalens, confirming the successful realization of the designed nanostructures. Additional details are provided in Section S5 (Supporting Information). The measured focal spot intensity distributions at the wavelength of 632.8 nm show excellent agreement with simulation predictions, as demonstrated in Figure 3b,c. The measured focusing efficiency are 30% and 60% under x and y polarized incident light at the design wavelength of 632.8 nm, respectively. The focusing efficiency at off‐design wavelengths is provided in Section S8 (Supporting Information). While the metalens exhibits lower focusing efficiency compared to a conventional refractive lens, this can be compensated for by adjusting the source power to enhance the incident intensity, thereby enabling the detection of a satisfactory return signal in our application. The actual axial focal position distribution was determined through calibration, allowing us to establish the relationship between wavelength and focal position (displacement). Subsequently, precise measurements were conducted using a calibrated curve, with the calibration experimental setup. The CCS‐ML measurement system consists of a fiber‐coupled white light‐emitting diode (LED) source (Thorlabs, MWWHF2) covering a wavelength range of 500–700 nm, a spectrometer (Ocean optics, HR6) with 0.22 nm resolution for spectral detection, and a chromatic confocal probe comprising a parabolic reflective mirror (Lbtek, RFCAL‐1.8‐APC) and the metalens. All components are interconnected via a three‐port multimode fiber circulator (Thorlabs, WMC1H1S) with a core diameter of 50 µm, exhibits a pinhole‐like filtering effect. A plane mirror serves as the target object, positioned on a motorized precision translation stage that allows movement along the optical axis. To account for motion errors inherent in the precision stage, a picometer interferometer records the mirror's exact displacement while remaining coaxial with the optical axis to eliminate Abbe errors and cosine errors.

During calibration, the plane mirror moved axially in equal increments of 1% of the full measurement range (4 µm for x‐pol and 15 µm for y‐pol modes), collecting spectral signal data at 110 positions to cover slightly more than the full measurement range, with each position measured five times and averaged. The acquired spectral signal data was processed using wavelet transform method for noise reduction, followed by Gaussian function fitting to extract the peak position of spectral within a defined acquisition window. Figure 3e presents the measured spectral signals at five locations, showing that the x‐pol mode with its higher NA produces narrower spectral bandwidths, corresponding to better axial resolution compared to the y‐polarization mode. The obtained results of peak wavelength and displacement were fitted using nonlinear least‐squares algorithm based on the following curve model:

(2) $$l\left(\lambda ;\beta \right)=\;\;a_{0}+\sum_{n=1}^{6}a_{n}\cos\left(n\omega \lambda \right)+b_{n}\sin\left(n\omega \lambda \right)$$

where $\beta ={\left(a_{0},a_{1},\text{…},b_{6},\omega \right)}^{T}\;$ represents the vector of parameters to be fitted, and *λ* denotes the measured peak wavelength. The optimization objective is to minimize the sum of squared residuals:

(3) $$\hat{\beta }=\arg\min S\left(\beta \right)$$

(4) $$S\;\left(\beta \right)=\sum_{i=1}^{m}{\left[l_{i}-l\left(\lambda_{i};\beta \right)\right]}^{2}$$

where $\hat{\beta }\;$ are denoted as the fitted parameters, representing the optimized solution vector obtained through nonlinear least‐squares regression, *m* indicates the total number of measurement points. The calibration curves for two modes are presented in Figure 3f, as evidenced by root‐mean‐square (RMS) errors of 0.12 and 0.69 µm, respectively. The calibration curve under x‐polarization is in excellent agreement with the simulation results presented in Figure 2h. For y‐polarization, the calibrated curve exhibits a 100 µm extension compared to the simulation results in Figure 2i. This shift in the theoretical dispersion curve is attributed to the non‐uniform intensity profile of the light source combined with the long depth of focus of the metalens. A detailed discussion is provided in Section S10 (Supporting Information).

### 2.4 Axial Measurement Accuracy and Resolution

**Figure 4:**
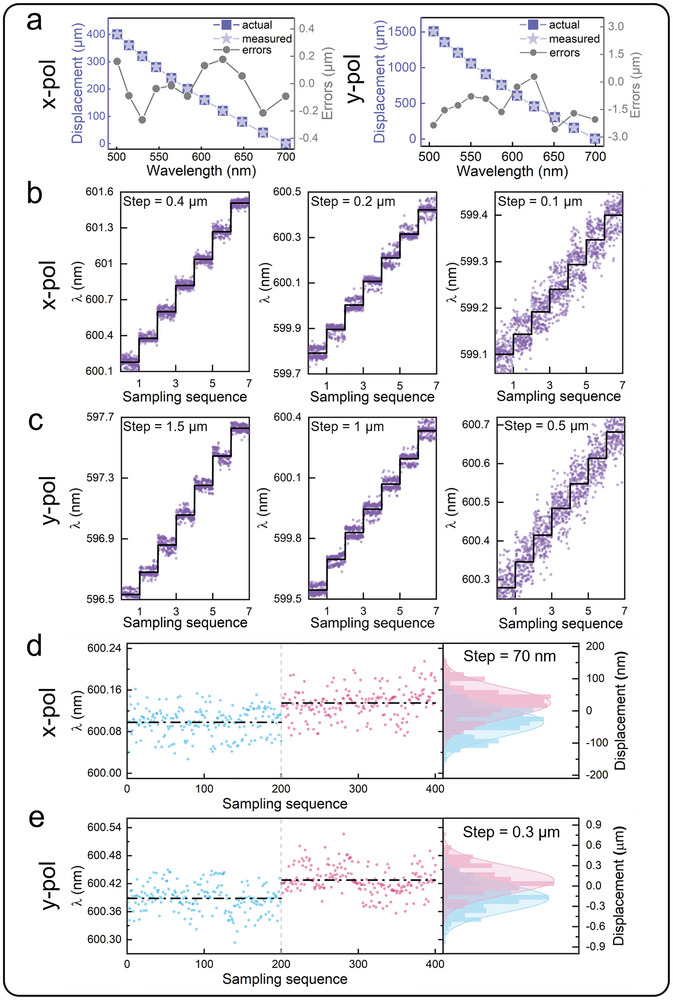
a) The measured axial accuracy of CCS‐ML. b) The measured peak wavelength at different measurement steps of 0.4 µm (left), 0.2 µm (center), and 0.1 µm (right) for x‐pol mode. c) The measured peak wavelength at different measurement steps of 1.5 µm (left), 1 µm (center), and 0.5 µm (right) for y‐pol mode. d,e) Statistics of the measurement peak wavelength at different positions for x‐pol and y‐pol modes. The two positions are just resolved and the step sizes are 70 nm (d) and 0.3 µm (e).

Following CCS‐ML calibration, axial accuracy measurements were performed using the identical optical system configuration shown in Figure 3d. A high‐precision displacement stage executed scans across the measurement range with step intervals of 40 and 150 µm under both x‐ and y‐polarized illumination, respectively, while a picometer interferometer provided actual displacement measurements. The measurement displacements were derived from the calibrated curve, with axial accuracy defined as the deviation between measured and actual displacements. As presented in **Figure** **4**a, it can be derived that the system achieved accuracy levels: x‐pol mode measurements maintained [‐0.3, 0.2] µm, and y‐pol mode measurements exhibited [‐2.6, 0.3] µm accuracy bounds across the full measurement range.

The calibration curve of the CCS‐ML measurement system demonstrates an approximately linear relationship between the peak wavelength and axial measurement position. Therefore, the theoretical axial resolution is estimated using the following expression:

(5) $$Re=\frac{\Delta L}{\Delta \lambda }\cdot \delta$$

where *Re* represents the axial resolution, Δ*L* represents the full measurement range, Δλ indicates the working wavelength range, δdenotes the resolution of the spectrometer. Based on Equation (5), the estimated theoretical axial resolutions for the x‐pol and y‐pol modes are 0.44 and 1.73 µm, respectively. To accurately quantify the true axial resolution of the CCS‐ML measurement system, the motorized precision translation stage used in the calibration experiments was replaced with a piezoelectric translation stage. The piezoelectric stage offers enhanced precision and stability, allowing for more accurate measurements of small displacement. Figure 4b illustrates the test results for the CCS‐ML measurement system under x‐pol mode near the central wavelength of 600 nm, with varying measurement step sizes. Each red dot represents a peak wavelength obtained from a single measurement. The three subplots include black horizontal lines corresponding to the displacement amounts when the piezoelectric platform moves in increments of 0.4, 0.2, and 0.1 µm. Figure 4c shows the test results for the CCS‐ML measurement system under y‐pol mode, again with different measurement step sizes of 1.5, 1, and 0.5 µm. The experimental results clearly demonstrate that the measurement system is able to distinctly resolve differences in peak wavelength when step sizes of 0.2 and 1 µm for x‐pol and y‐pol modes. However, as the step size decreases to 0.1 and 0.5 µm, fluctuations in the light source's stability cause the peak wavelengths to overlap within the adjacent step, leading to ambiguity in resolution.

To achieve optimal performance of the CCS‐ML measurement system, we systematically performed repeated displacement measurements using progressively smaller step sizes. At each step position, we acquired 200 consecutive peak wavelength measurements to establish statistical distributions, and compared the differences in peak wavelengths obtained from two consecutive measurements. We assume that the statistical results of the measured peak wavelengths follow a Gaussian distribution $\lambda_{i}∼N\left(\mu ,\sigma \right)$, λ_*i*_ represents the peak wavelength, which is independent and unrelated, μ and σ are the mean and standard deviation of a group of measurement data, *i* represents the number of measurements. When $\bar{\Lambda_{2}}-\;\bar{\Lambda_{1}}=0.675\sigma_{1}+0.675\sigma_{2}$, the probability that $\lambda_{i}\in \Lambda_{1}$ or $\lambda_{i}\in \Lambda_{2}$ is 50%, it is just resolved, where $\bar{\Lambda }$ is the average value of the peak wavelengths for each group of measurements.^[^ ^35^ ^]^ Figure 4d,e presents the Gaussian distribution fitting results of measured peak wavelengths for x‐pol and y‐pol modes, respectively. The minimum resolvable step for x‐pol mode is 70 nm, where $\bar{\Lambda_{1}}=600.098\;\mathrm{nm},\bar{\Lambda_{2}}=600.1353\;\mathrm{nm},\;\sigma_{1}=0.0273\;\mathrm{nm},\sigma_{2}=0.0286\;\mathrm{nm}$, and the minimum resolvable step for y‐pol mode is 0.3 µm, where $\bar{\Lambda_{1}}=600.3885\;\mathrm{nm},\bar{\Lambda_{2}}=600.4284\;\mathrm{nm},\;\sigma_{1}=0.0296\;\mathrm{nm},\sigma_{2}=0.0297\;\mathrm{nm}.$

### 2.5 Measurement Experiment

**Figure 5:**
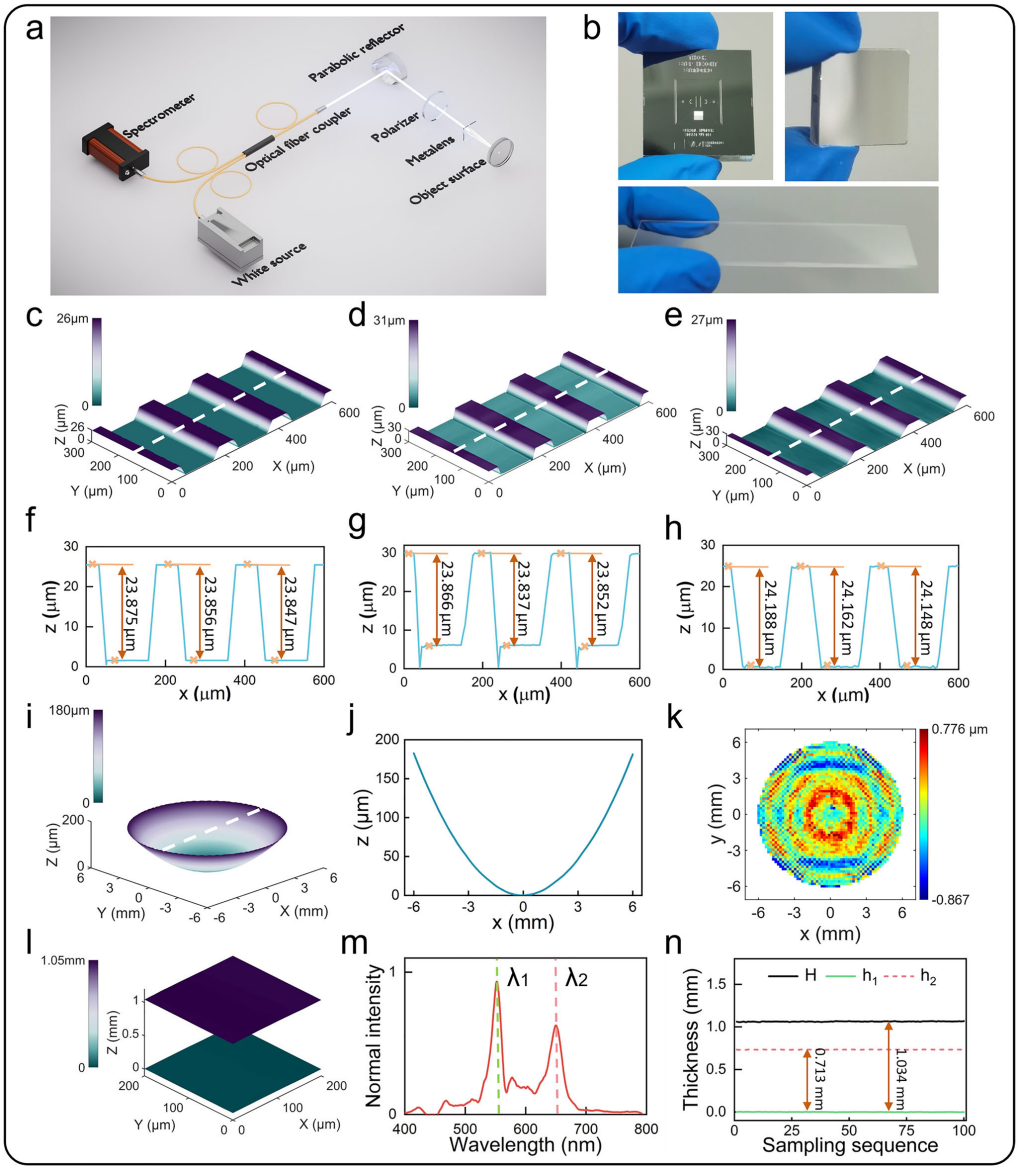
a) Optical measurement setup of CCS‐ML. b) Diagram of the sample under test, including a standard step block, a concave spherical reflective mirror and a transparent glass slide. c–e) The measured 3D surface topography of standard step block by white‐light interferometry (c), CCS‐ML x‐pol mode (d), CCS‐ML y‐pol mode (e). The measurement areas are 600 × 300 µm^2^, 600 × 300 µm^2^, and 600 × 300 µm^2^ respectively. f–h) The cross‐sectional profiles along the white dashed lines in Figure 5c–e. i,j) The measured 3D surface topography of concave spherical reflective mirror by CCS‐ML x‐pol mode and the cross‐sectional profiles along the white dashed lines. k) The measured errors of the concave spherical reflective mirror. l) The measured 3D surface topography of 200  ×  200 µm^2^ area for transparent glass slide by CCS‐ML y‐pol mode. m) The measured spectral signals of transparent glass slide. λ_1_ and λ_2_ represents the wavelength of reflected light from the lower and upper surface. n) Diagram of the measured thickness of transparent glass slide at one point. h_1_ and h_2_ correspond to the positions of λ_1_ and λ_2_. H is the thickness of transparent glass slide.

We conducted a series of 3D topography measurements to validate the performance of our CCS‐ML measure system. **Figure** **5**a illustrates the experimental optical setup, while Figure 5b shows the test samples, including a standard step block (SHS‐24.0QC), a concave spherical reflective mirror, and a transparent glass slide. To verify the measurement accuracy, we first characterized the standard step height. Figure 5c–e presents the 3D surface topography obtained using: white light interferometry, CCS‐ML x‐pol mode and CCS‐ML y‐pol mode. The standard step block was fabricated using an etching process, resulting in adjacent steps that exhibit slopes and depressions. Our measure system was able to clearly measure these variations though we are more focused on the height difference of the steps. Compared to the x‐pol mode, the y‐pol mode exhibits spurious signals at the baseline recess. This artifact arises due to the varying inclination angles of the recess in combination with the low numerical aperture (NA) of the y‐pol mode. This effect is analogous to the batwing effect observed in white‐light interferometry when measuring steps. In our analysis, these spurious signals are treated as invalid data and are removed. Figure 5f–h shows the cross‐sectional profiles along the white dashed lines in Figure 5c–e: The results of white light interferometry are 23.875, 23.856, 23.847 µm, the results of CCS‐ML x‐pol mode are 23.866, 23.837, 23.852 µm, and the results of CCS‐ML y‐pol mode are 24.188, 24.162, 24.148 µm. The results demonstrate excellent agreement between all three methods that confirms our CCM sensor achieves high accuracy.

In practical measurement applications, selecting an appropriate performance sensor requires careful consideration of the trade‐off between measurement range and resolution. To demonstrate the switchable capability and operational convenience of our CCS‐ML measure system, we conducted measurements on both a concave spherical mirror and a transparent glass slide. For the concave spherical mirror (area 12 mm×12 mm, nominal radius 100 mm), the x‐pol mode provided the necessary high resolution and tilt measurement capability. Figure 5i shows the 3D surface topography of a circular measurement area with a diameter of 12 mm. Figure 5j presents the cross‐sectional profile along the white dashed line in Figure 5i. The fitted radius of curvature 100.025 mm was obtained through least‐squares algorithm according to the measured data. Figure 5k shows the measured errors of the concave spherical mirror, and the errors fall within [‐0.867, 0.766] µm. Main sources of measurement error include: wavelength drift during measurements and straightness error of movement stage. For transparent glass thickness measurement, the y‐pol mode's extended range enabled characterization of a 1 mm thick glass slide. Figure 5l shows the 3D morphology map of a 200 µm×200 µm^2^ area on a 1 mm thick transparent glass sheet. When light focuses on both the upper and lower surfaces of the glass, the corresponding wavelengths are captured by the spectrometer, as depicted in Figure 5m. λ_1_and λ_2_ represents the reflected light from the lower and upper surface, and the height difference between λ_1_and λ_2_ can be calculated as Δ*h*  =  *l*(λ_1_) − *l*(λ_2_). Due to the refraction occurring at the air‐glass interface, the actual glass thickness H can be approximated by $H=\frac{n_{2}}{n_{1}}\Delta h$ for small incident angles, where *n*_2_ =  1.45 represents the refractive index of glass and *n*_1_ =  1 denotes the refractive index of air. Figure 5n presents the statistical results of 100 repeated thickness measurements at a single point, yielding a mean glass thickness of 1.034 mm, which demonstrates excellent agreement with 1.033 mm measured using a digital micrometer. The results validate our measure system ability to address diverse measurement scenarios through its unique polarization‐multiplexed design while maintaining compact form factor and operational simplicity.

## 3 Conclusion 

We have developed an advanced chromatic confocal sensor system with switchable measurement capabilities that offers significant advantages for high‐precision metrology applications. The key innovation lies in our polarization‐multiplexed design that integrates two distinct measurement modes within a single compact probe. Compared to conventional chromatic confocal systems requiring complex multi‐lens assemblies, our simplified optical architecture achieves comparable < 100 nm resolution while dramatically reducing hardware complexity and system footprint. The polarization dimension of light provides an additional measurement mode, which significantly reduces both cost and operational complexity when confronted with diverse measurement tasks.

Metasurfaces demonstrate inherent and pronounced dispersion characteristics that are fundamentally superior to those of conventional refractive lenses, thereby enabling substantial simplification of optical system architectures. When compared to traditional diffractive optical elements, metasurfaces exhibit enhanced signal‐to‐noise performance and diffraction efficiency while offering multidimensional light manipulation capabilities. These distinctive properties confer significant advantages in terms of system integration flexibility and multifunctional operation. During the calibration process, we established a one‐to‐one correspondence between peak wavelength and displacement distance, effectively creating an optical ruler. Within the wavelength range of 500–700 nm, the measurement ranges for x‐pol mode and y‐pol mode are 400 µm and 1.57 mm, the axial accuracy is ± 0.25 and ± 1.45 µm, respectively. The axial resolution experiment demonstrated the minimum scale of this optical ruler, revealing that the optimal axial resolutions for x‐pol and y‐pol modes near the central wavelength of 600 nm could reach 70 nm and 0.3 µm, respectively. Finally, in our measurement experiments, including measurements of a standard step block, a concave spherical mirror, and a glass sheet, conclusively demonstrates the robust practical performance and measurement capabilities of our CCS‐ML measure system in real‐world applications. It is worth noting that compared to previously reported metasurface‐based confocal measurement system, our CCS‐ML achieves higher measurement accuracy and resolution, as summarized in Table S1 (Supporting Information).

This study demonstrates the significant potential of metasurfaces to act as a transformative force in the evolution of traditional measurement devices toward more integrated and versatile systems. Future research will focus on leveraging the powerful light‐manipulation capabilities of metasurfaces to further expand the sensor's measurement modalities. We will also investigate metasurface‐fiber integration strategies to realize more compact and robust system architectures, aiming to address the challenges of measurements in highly confined spaces. In conclusion, this work demonstrates the significant potential of metasurface technology in ultraprecision metrology applications, effectively paving the way for their transition from laboratory research to practical engineering deployment.

## 4 Statistical Analysis

In the axial resolution measurement experiment, a mirror was mounted on a piezoelectric stage with a resolution of less than 5 nm and moved in fine steps. At each position, the returning spectral signal was recorded by a spectrometer. The measured spectral signal *I*(λ) was normalized according to the formula *I*/*I_max_*, and the peak wavelength $\lambda_{M}^{i}$ was determined using a wavelet transform method and peak extraction algorithm, where M denotes the index of each group, *i* denotes the index of one group. For each position, a histogram was generated from the resulting data points, with the number of bins set to $\sqrt{n}$, where *n* is the number of data points measured at that position, and […] is rounded to the nearest integer. The data were fitted using a Gaussian distribution function, from which the mean ${\bar{\Lambda }}_{M}$ and standard deviation (SD) σ_*M*_ of the fit were obtained. The region around the central wavelength of 600 nm was repeatedly measured with a step size of 70 nm and 0.3 µm for x‐pol and y‐pol, respectively. The positional differences retrieved from the two sets of measurement data were then observed. The $\bar{\Lambda_{1}}$ with SD σ_1_ and $\bar{\Lambda_{2}}$ with SD σ_2_ are the normalized fitting coefficients center of Gaussian fitting of the two‐measurement data, respectively. Then, $\lambda_{1}^{i}∼N\left(\bar{\Lambda_{1}},\sigma_{1}\right)$ and $\lambda_{2}^{i}∼N\left(\bar{\Lambda_{2}},\sigma_{2}\right)$ can be obtained. The extent of the overlapping region between the two datasets depends on $\bar{\Lambda_{2}}-\bar{\Lambda_{1}}.$ When $\bar{\Lambda_{2}}-\;\bar{\Lambda_{1}}=0.675\sigma_{1}+0.675\sigma_{2}$, the probability that $\lambda_{1}^{i}\in \Lambda_{1}\;\mathrm{or}\;\lambda_{1}^{i}\in \Lambda_{2}\left(\mathrm{similary}\;\lambda_{2}^{i}\in \Lambda_{1}\;\mathrm{or}\;\lambda_{2}^{i}\in \Lambda_{2}\right)$) is 50%, the displacement is just resolved.

## Conflict of Interest

The authors declare no conflict of interest.

## Supporting information



Supporting Information

## Data Availability

The data that support the findings of this study are available from the corresponding author upon reasonable request.
