# Supplementary material for: Polarization‐Multiplexed Metalens Enables Switchable and Compact Chromatic Confocal Sensing with Dual‐Mode Precision Control
Source: Adv Sci (Weinh). 2025 Nov 12;13(6):e17093. doi: 10.1002/advs.202517093 (PMC12866683; doi:10.1002/advs.202517093)
Supplement: Supplementary file 1 — Supporting Information [file ADVS-13-e17093-s001.docx]

**Supplementary Information for**

**Polarization-Multiplexed Metalens Enables Switchable and Compact Chromatic Confocal Sensing with Dual-Mode Precision Control**

Zhicheng Zhao^†^, Yuting Jiang^†^, Tao Lai, Wenxiang Peng, Yueqiang Hu^﹡^, and Shanyong Chen^﹡^

**^*^Corresponding authors**: **Shanyong Chen**, College of Intelligence Science and Technology, National University of Defense Technology, Changsha 410073, China, Email: [mesychen@163.com;](mailto:mesychen@163.com;d) **Yueqiang Hu**, College of Mechanical and Vehicle Engineering, Hunan University, Changsha 410082, China, Email: [huyq@hnu.edu.cn](mailto:huyq@hnu.edu.cn).

**Zhicheng Zhao, Tao Lai, Wenxiang Peng:** College of Intelligence Science and Technology, National University of Defense Technology, Changsha 410073, China.

**Yuting Jiang:** College of Mechanical and Vehicle Engineering, Hunan University, Changsha 410082, China

^†^These authors contributed equally to this work

**Section 1: Optical constant of SiN.**

Figure S1 shows the experimentally measured *n* and *k* values of silicon nitride (SiN) in the 200 nm -1000 nm band.


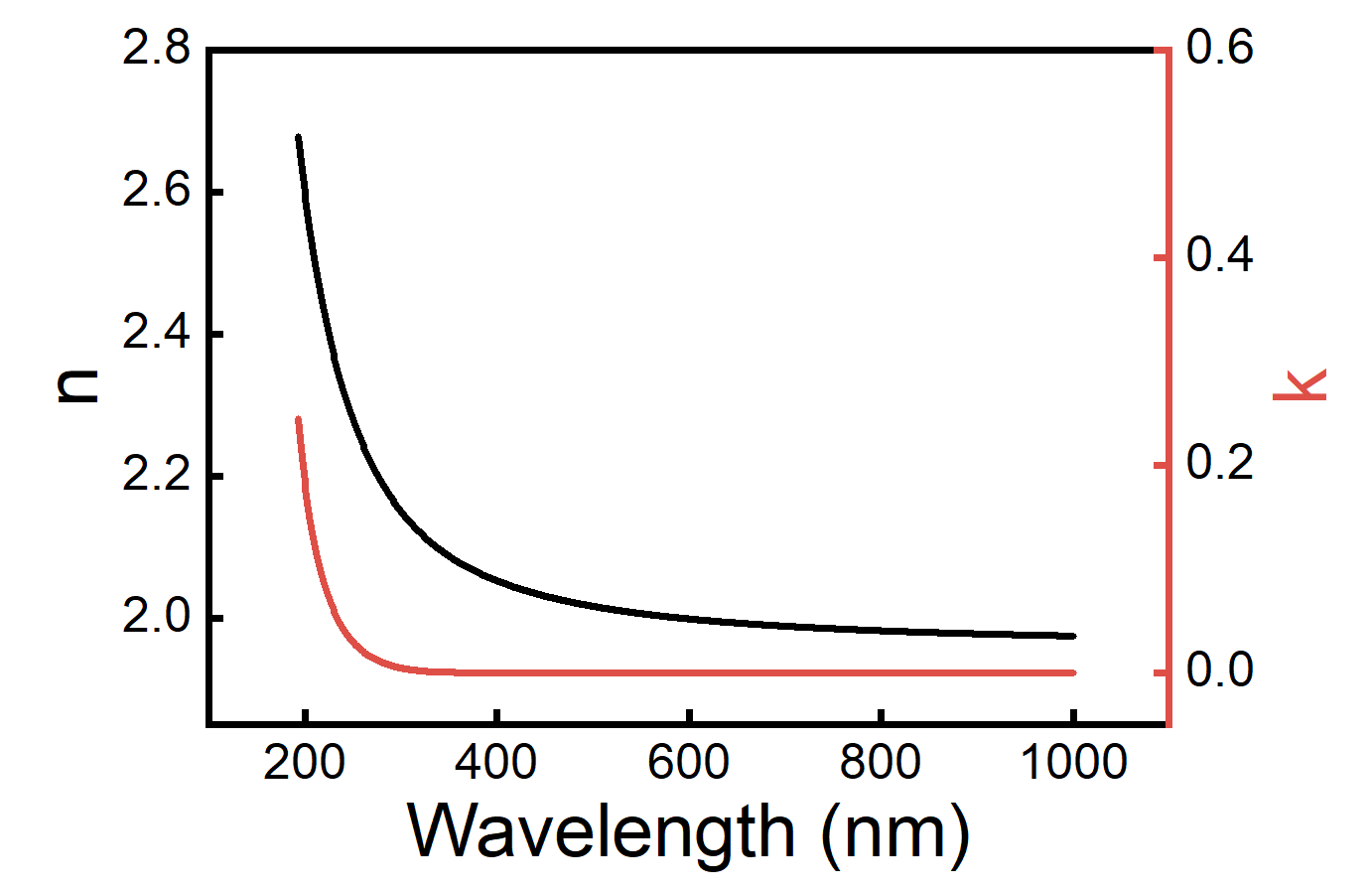


**Figure S1.** The Optical constant of SiN.

**Section 2: Theoretical derivation of wavelength and effective refractive index.**

The formula S1 expresses the relationship between the wavelength and the effective refractive index (ERI) in the one-dimensional equivalent medium theory.

$n_{eff}={(\frac{1-F}{n_{air}^{2}}+\frac{F}{n_{SiN}^{2}})}^{-\frac{1}{2}}$ (S1)

where 𝐹 is the duty cycle, $n_{eff}$ is the effective refractive index, $n_{air}$ is the refractive index of air, $n_{SiN}$ is the experimentally measured refractive index of silicon nitride (SiN). Figure S2 shows the relationship between the wavelength and the effective refractive index derived from the formula S1 when 𝐹=0.5. It can be observed that within the wavelength range of 500 - 700 nm, the structural dispersion of silicon nitride is very small.


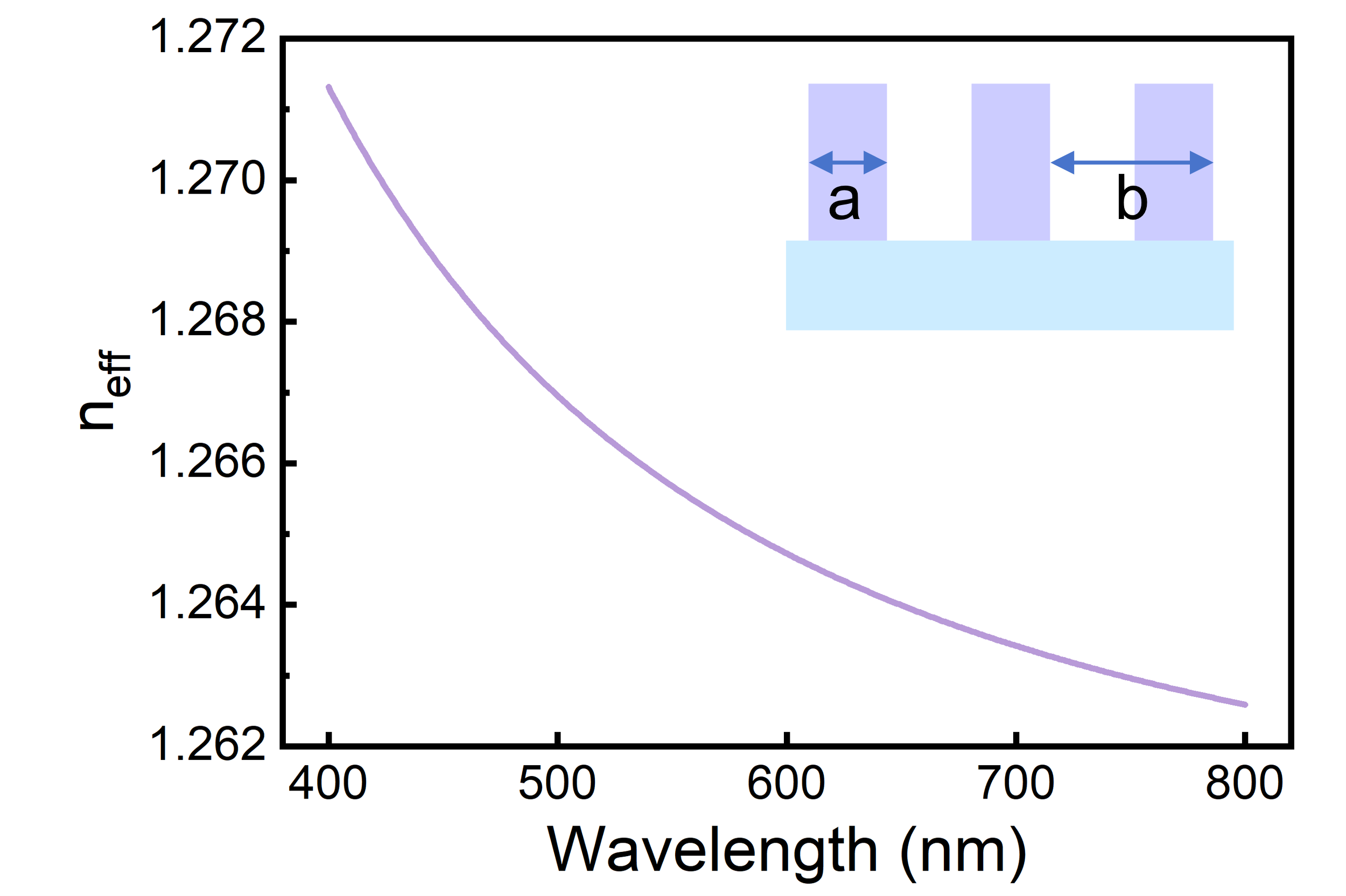


**Figure S2.** The relationship between the wavelength and the effective refractive index is derived from the one-dimensional equivalent medium theory

**Section3: The intensity profile of source.**

The LED's intensity profile, provided in the Figure S3, exhibits a convex distribution across the 500-700 nm band.


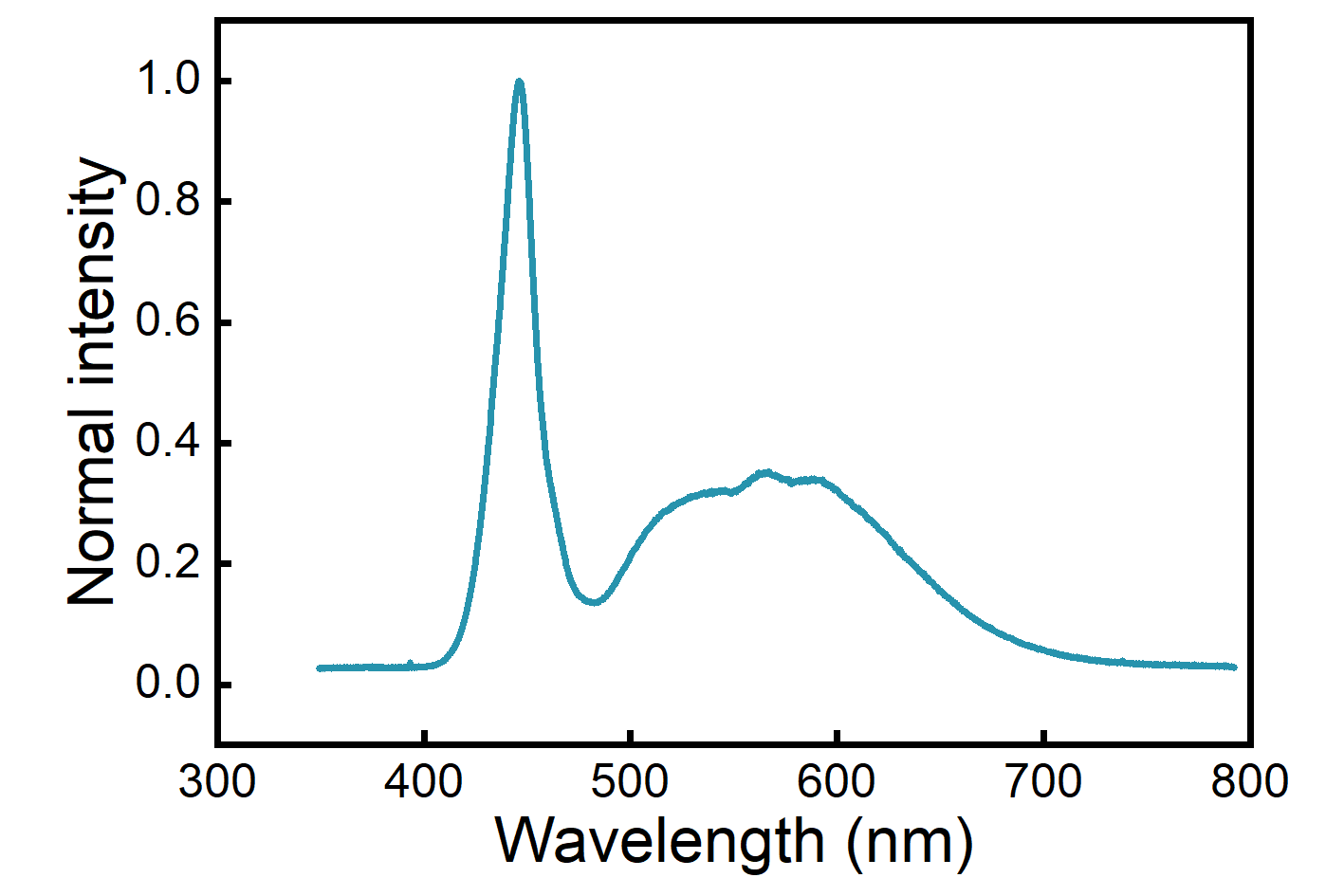


**Figure S3.** The intensity distribution of source.

**Section 4: Device fabrication.**

The manufacturing process is shown in the Figure S4. First, the electron-beam resist (EBR) was spin-coating at 3000 rpm on the 1200-nm-thick silicon nitride film on the quartz substrate and baked on a hot plate for 1 min at 180 ℃. Then, the sample was exposed by electron-beam lithography (EBL) with a 100-KV voltage and a beam current of 200 pA. Later, we used the atomic layer deposition (ALD) system to deposit a layer of chromium approximately 20 nm thick, followed by chromium lift-off. Next, the reactive-ion-etch (RIE) was applied to etch through the 1200-nm-thick silicon nitride layer. Finally, we removed the remaining EBR and the silicon nitride structures with a high aspect ratio are obtained.


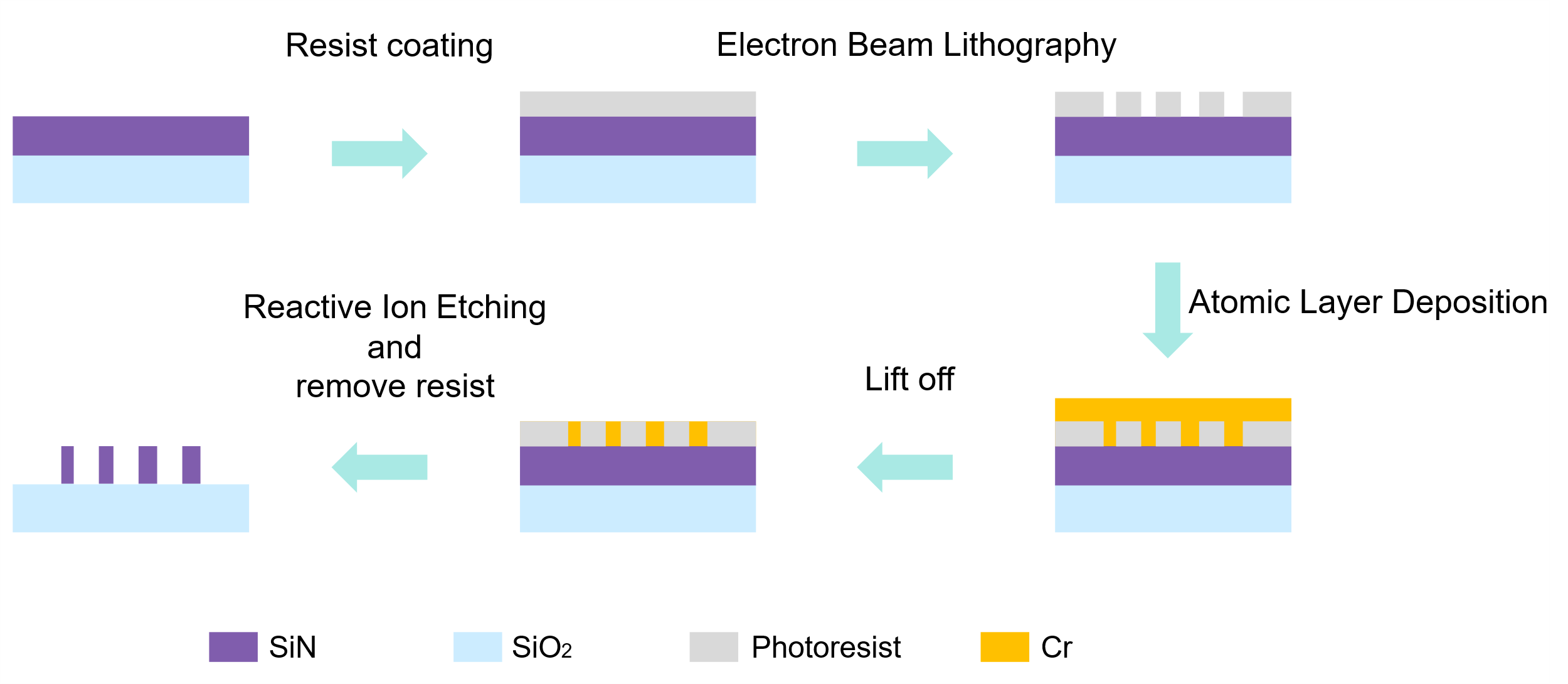


**Figure S4.** The fabrication process of SiN structure.

**Section 5: Scanning electron microscopy image of the fabricated metalens**

Figure S5 a-b shows the scanning electron microscopy image of the fabricated metalens. As can be seen in the Figure S5 a, the width range is 100 to 300 nm. Figure S5 b shows the edge part of the metalens, which is tilted at 45 degrees. According to this document, at a 100% viewing scale, a measurement of 12 mm on the image corresponds to an actual physical length of 2 μm. Here, we denote the ratio of the actual length to the image length as R, the height of meta-atom on the image as h, the actual physical height as H. Then we can calculate the actual physical height of meta-atom by $H=\sqrt{2}\times h\times R$. Substituting h = 5.1mm, R = 0.000167, we can obtain H = 1.2 μm. Due to the poor electrical conductivity of silicon nitride, a 5nm chromium coating was applied before scanning electron microscopy. It is noted that the flaky particulates present on the nanofin in the picture, which was caused by the chromium.





**Figure S5.** (a) The scanning electron microscopy image of the fabricated metalens


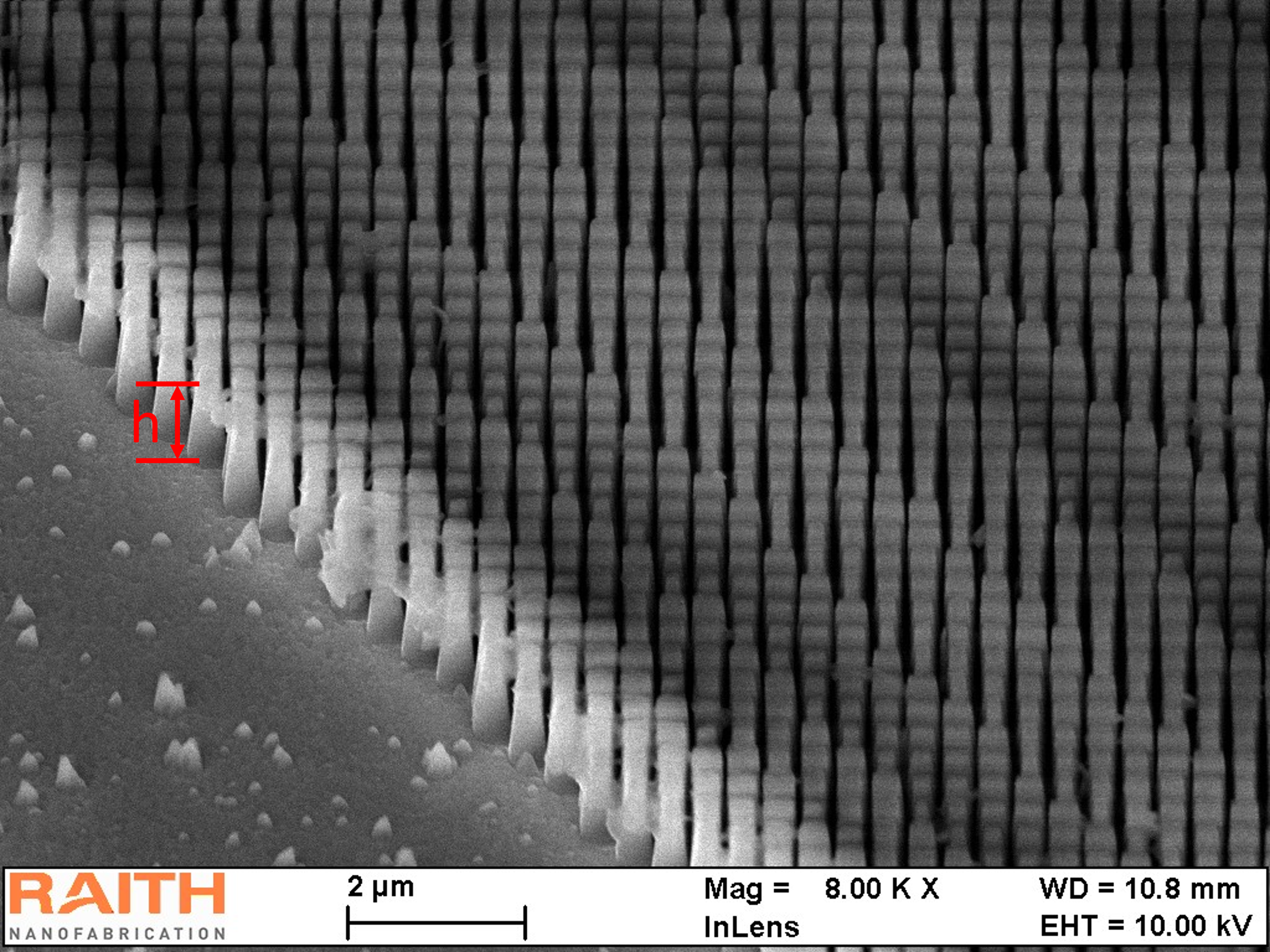


**Figure S5.** (b) The scanning electron microscopy image of the fabricated metalens (side-view).

**Section 6: The effects of processing errors.**

For the processing plan used in this article, we had selected two locations where errors are most likely to occur and performed corresponding simulations. First, we simulated the loss of the structure height during the etching process. We uniformly reduced the height of all nanostructures by 200 nm. The resulting intensity distributions on the focal plane and the xz-plane are shown in Figures S6 b and e, respectively. Compared to the optical field distribution of the ideal structures (Figures S 6 a and d), the reduction in height has a minimal impact. This is because the nanostructures at the reduced height of 1000 nm still achieve a full 0–2π phase response, allowing the metalens to maintain the designed phase gradient profile. The original height of 1200 nm provides a sufficient fabrication margin to accommodate such process variations. We then simulated the optical field distribution under deviations in the lateral dimensions of the nanostructures from their design values. By introducing uniformly distributed random errors within a range of ±40 nm to the nanostructures (simulation results shown in Figures S6 c and f), we observed that although the focal position shift is minimal, the lateral dimensional errors lead to a significant degradation in focusing intensity

**
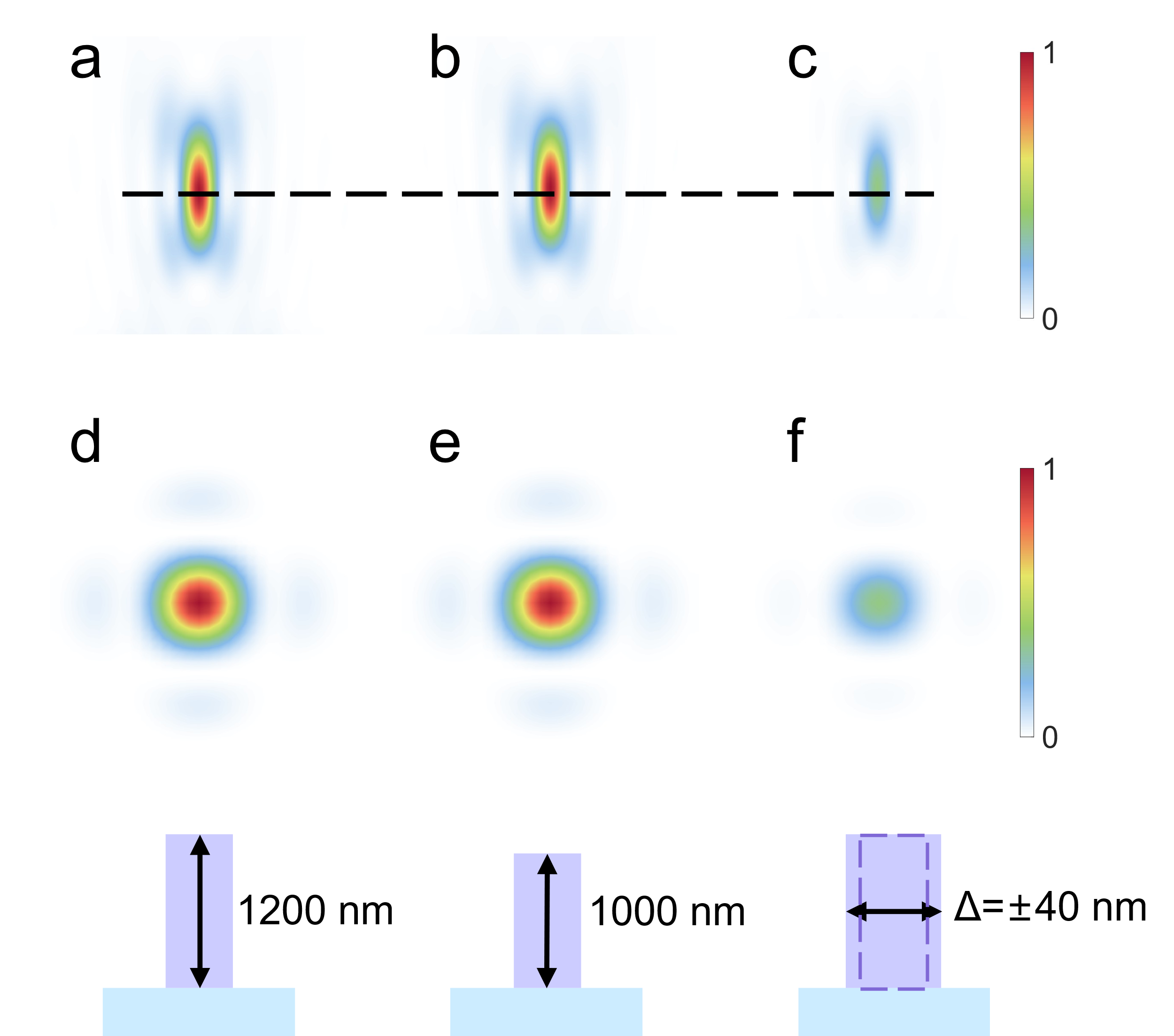
**

**Figure S6.** Simulation of the impact of overetching and lateral dimensional errors on the focusing effect

**Section 7: Optical characterization.**

The optical setup used to verify the metalens is shown in Figure S7. The collimated laser beam was directed through a polarizer onto the metalens sample. Then, the laser beam is imaged on the CCD through the metalens and the objective lens. Finally, the translation stage moved from the position where the sample surface is imaged, and the camera acquired an imaging pattern every 1 μm until it stops at 200 μm from the starting position.


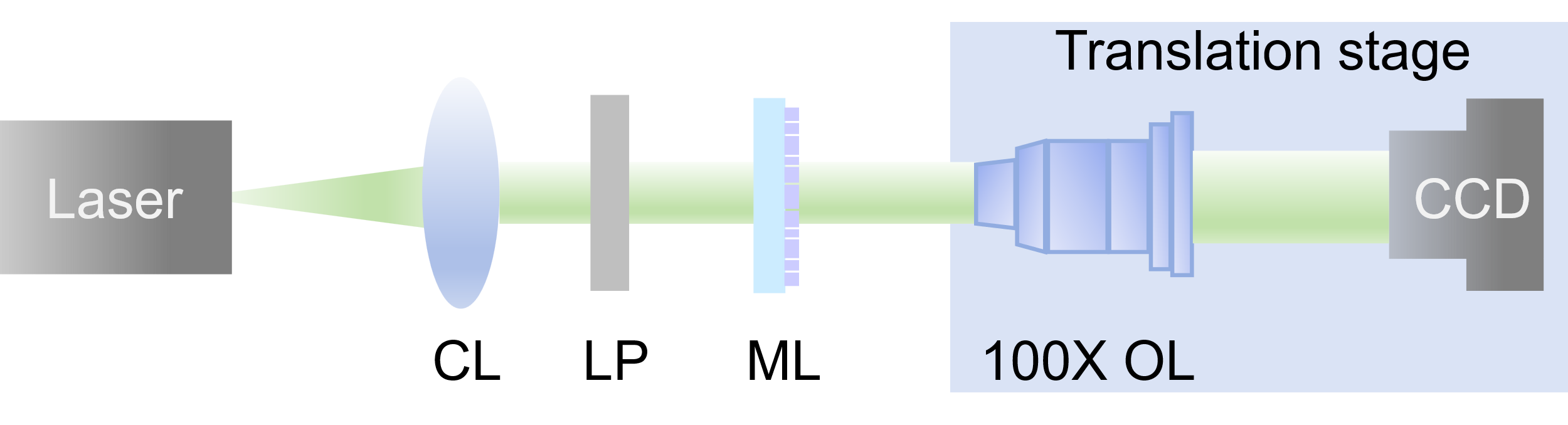


**Figure S7.** The Schematic of optical characterization system. CL: collimating lens. LP: linear polarizer. ML: metalens. OL: objective lens.

**Section 8: Focusing efficiency calculation method.**

The focusing efficiency of the matching metalens within the 500-700nm range is calculated using the scalar diffraction algorithm as shown in Figure S8, where the focusing efficiency is defined as the intensity within three times the full width half height (FWHM) at the focus plane divided by the intensity of incident light within the range of the metalens. Due to the spherical aberration, the focusing efficiency decreases at non-design wavelengths.


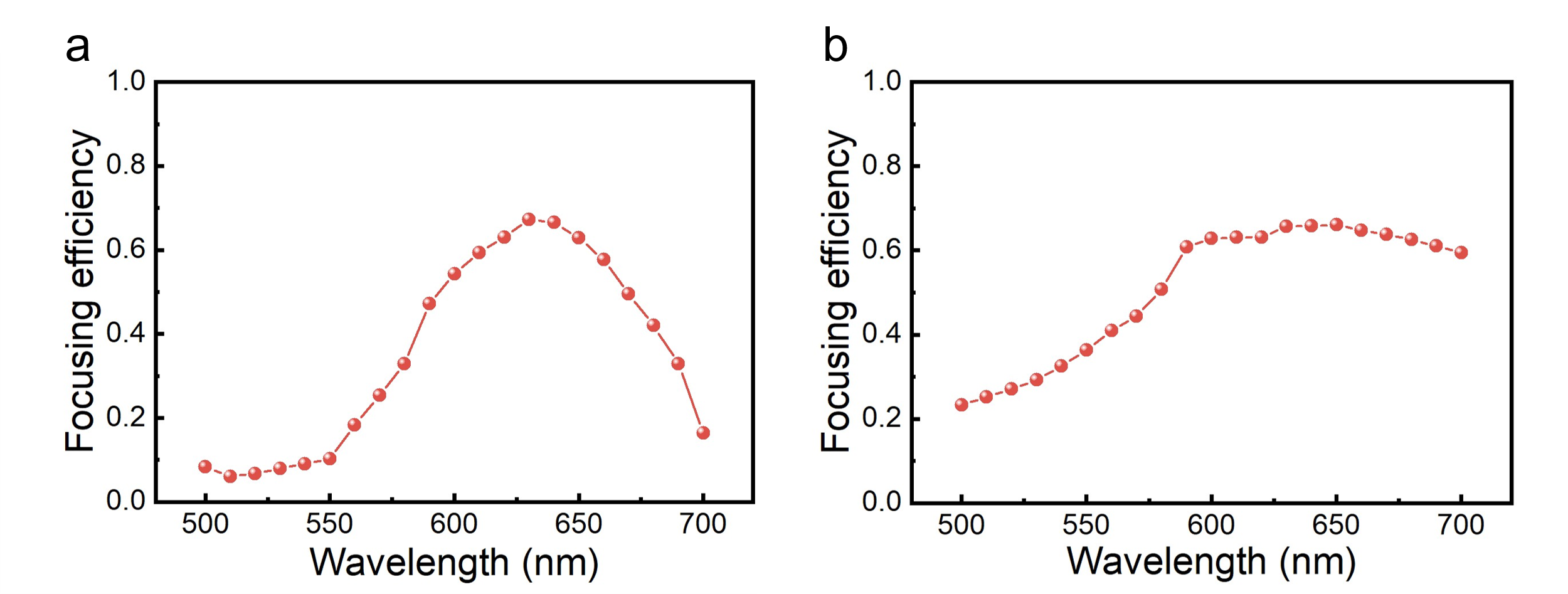


**Figure S8.** Focusing efficiency. (a) The focusing efficiency of the metalens under x-polarized incident light. (b) The focusing efficiency of the metalens under y-polarized incident light.

**Section 9: Comparative analysis of theoretical and experimental focal lengths.**

The focal point is defined as the position of the maximum intensity, as shown in the one-dimensional plot of Figure S9 a-b. The focal positions derived from the ideal phase profile (blue line), the matched phase profile (green line), and experimental measurements (red line), as illustrated in the Figure S9 c-d. In the initial design, the dispersion of the nanostructures was neglected. Under this assumption, light across the 500-700 nm band was assigned a uniform phase upon passing through the metalens, and the resulting focal position can be calculated using the following formula:

$$\varphi_{0}=-\frac{2\pi}{\lambda_{0}}(\sqrt{r^{2}+{f_{0}}^{2}}-f_{0})$$

$f=-\frac{\pi r^{2}}{\lambda\varphi_{0}}+\frac{\varphi_{0}\lambda}{4\pi}$ (S2)

where $r$ is the radius of metalens, $f_{0}$ is the focal length at design wavelength, $\lambda_{0}$ is design wavelength.

In the FDTD simulations, the phase and transmission responses of a unit structure were recorded across different wavelengths. The focal length for the matched phase profile was then calculated by incorporating this structural dispersion into the angular spectrum propagation method, using the following formula:

$$u_{z}\left( x,y \right)=\mathcal{F}^{-1}\left\{ \mathcal{F}\left\{ u_{0}\left( x_{0},y_{0} \right) \right\}\times H\left( f_{x},f_{y} \right) \right\}$$

$H\left( f_{x},f_{y} \right)=e^{ikz-i\pi\lambda z({f_{x}}^{2}+{f_{y}}^{2})}$ (S3)

where $u_{0}\left( x_{0},y_{0} \right)$ and $u_{z}\left( x,y \right)$ denote the complex amplitude distribution on the diffraction and observation screen, $H\left( f_{x},f_{y} \right)$ represents the diffraction transfer function, z is the propagation distance between the diffraction and observation screen.

As observed in Figure S9 c, the close alignment of the three curves indicates weak nanostructure dispersion under x-polarization, with minimal impact on performance, which also indirectly confirms that the nanostructure fabrication meets the design expectations. Similarly, the agreement between the blue and green lines in Figure S9 d suggests that dispersion effects remain negligible under y-polarization.


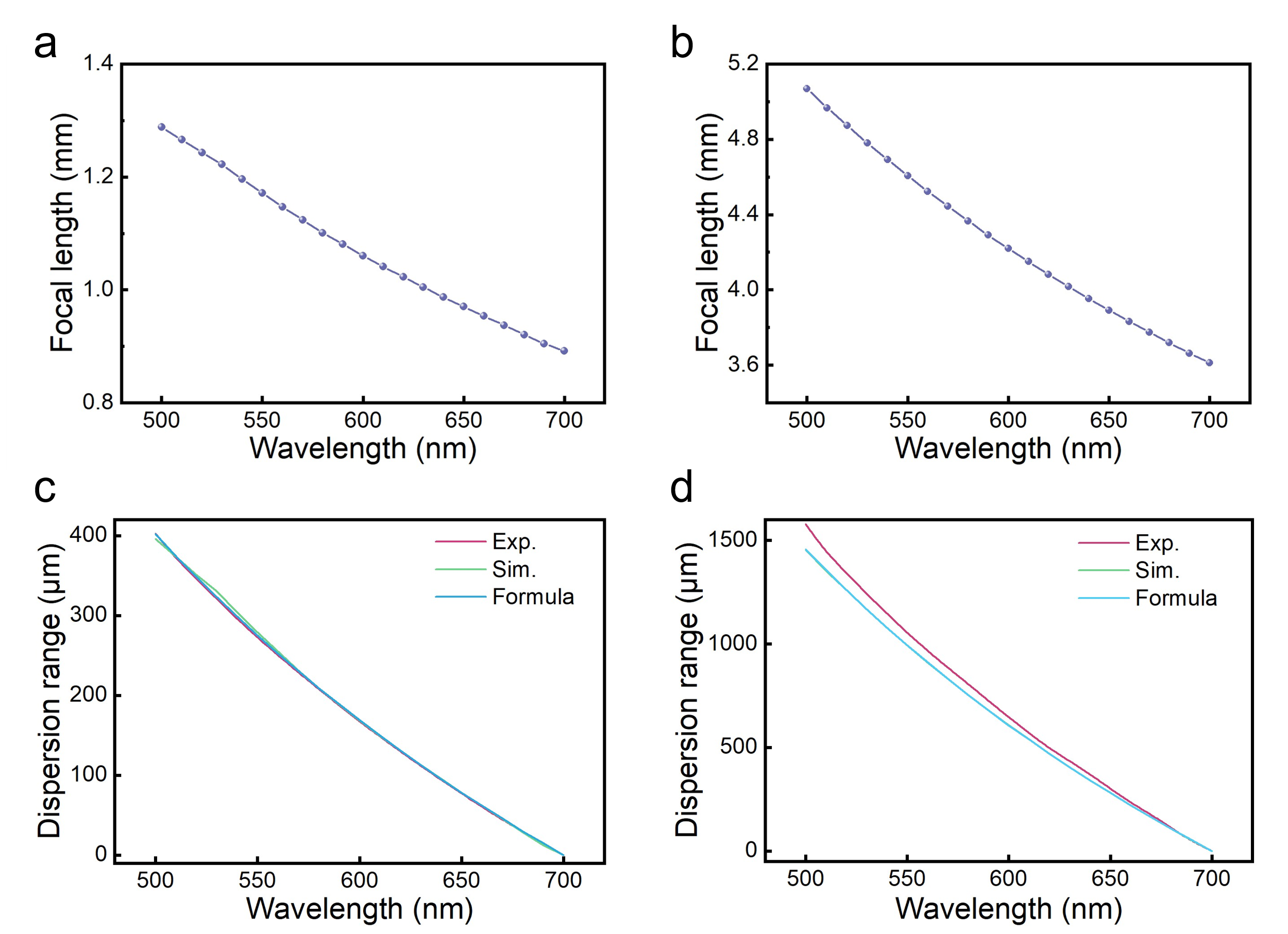


**Figure S9.** (a) The focal length of metalens under x-polarized incident light. (b) The focal length of metalens under y-polarized incident light. (c) The dispersion of metalens under x-polarized incident light. (d) The dispersion of metalens under y-polarized incident light. (“Exp.” is derived from experimental measurement, “Sim.” is derived from the angular spectrum propagation method, “Formula” is derived from the Formula S2.)

**Section 10: Effect of light source uniformity.**

It is noted that a deviation exists between the experimentally measured curve and the theoretical curves from Figure S10 d. This discrepancy is attributed to the non-uniform intensity profile of the LED source and the long focal depth of the metalens (NA=0.125). To illustrate this effect, consider the design wavelength of 632.8 nm, which has a theoretical focal point at 4 mm. However, the incident intensity at 630 nm is stronger than that at 632.8 nm. If the 4 mm position falls within the focal depth of 630 nm light, the stronger intensity at 630 nm will dominate the signal detected at that location, manifesting as a leftward shift of the theoretical focal curve.

A schematic diagram Figure S10 illustrates this mechanism. Using the peak LED intensity wavelength (565 nm) as a pivot, the theoretical curve (red line) shifts rightward for wavelengths below 565 nm (Figure S10 a) and leftward for wavelengths above it (Figure 10 b). Consequently, the overall measured dispersion across the 500-700 nm range is broader than theoretically predicted, as summarized in Figure S10 c.


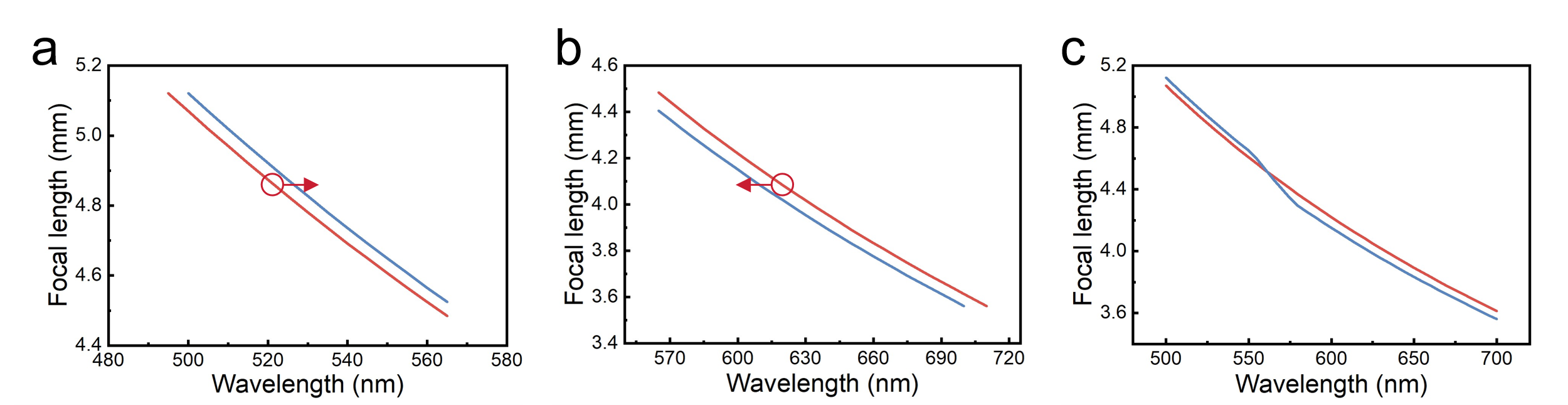


**Figure S10.** The effect of light source uniformity for focal length of metalens. Red line is the theoretical curve. Blue line is the actual curve.

**Section 11：Comparison of the measurement performance**

**Table S1** Performance comparison between metasurface-based chromatic confocal systems

| Working wavelength | Measuring range | NA | Axial accuracy | Axial resolution |  |
| --- | --- | --- | --- | --- | --- |
| 800-1000 *nm* | 1.56 *mm* | — | $\pm20 \mu m$ | — | [34] |
| 500-700 *nm* (x-pol) | 0.4 *mm* | 0.45 | $\pm0.25 \mu m$ | 70 *nm* | This |
| 500-700 *nm* (y-pol) | 1.57 *mm* | 0.125 | $\pm$1.45 *µ*m | 0.3 *µm* | work |

To the comparison with our CCS-ML, we further present a performance evaluation against existing metasurface-based system, as detailed in Table S1. The comparison highlights five key performance metrics that are critical for chromatic confocal design: working wavelength, measuring range, NA, axial accuracy, and axial resolution. It is essential to clarify the distinction between axial accuracy and axial resolution. Axial accuracy refers to the absolute positioning precision, while axial resolution is the minimum distance that can be distinguished along the *z*-axis. The table incorporates results from recent peer-reviewed publications.

Notably, the CCS-ML exhibits distinct advantages in terms of high axial accuracy and high axial resolution across the operational spectral range. Specifically, in terms of axial accuracy, under extended-range mode (y-pol), it offers an improvement of close to one order of magnitude within a comparable measuring range compare with Ref. 34. When switch to high-accuracy mode, the axial accuracy is higher. These results further highlight the potential of metalenses for enabling high-performance, lightweight, and multifunctional confocal sensing solutions.
